# Positive education for students’ mental health support

**DOI:** 10.1192/j.eurpsy.2022.1614

**Published:** 2022-09-01

**Authors:** R. Shilko, S. Egorov, Y. Zinchenko, V. Emelin

**Affiliations:** Lomonosov Moscow State University, Psychology, Moscow, Russian Federation

**Keywords:** positive education, psychological well-being, mental health

## Abstract

**Introduction:**

There has been obvious in university education the importance of mental health and psychological well-being of students along with academic achievements (Lambert et al., 2019). M. Seligman, one of the founders of positive psychology, characterizes positive education as education aimed at acquiring happiness along with knowledge and skills (Seligman et al., 2009).

**Objectives:**

The current study aims to overview the positive education impact on mental health and psychological well-being.

**Methods:**

Systematic analysis of research publications concerning positive education and its role for maintenance of mental health and psychological well-being.

**Results:**

While university administration collects student’s feedback on various aspects of the educational process, it almost never asks students for their opinion on what can be done for their psychological well-being. The promotion of psychological well-being among young people is becoming an increasingly popular topic, and positive education is increasingly emerging within education. Positive education can be seen as a general sphere of positive psychology and advanced practice in education that aims the development of students for both academic achievement and psychological well-being. Special attention in positive education is paid to the use of empirically proven methods and programs aimed at improving well-being, engagement in educational activities, optimism, positive emotions, life satisfaction and other positive experience.

**Conclusions:**

A particularly promising direction for the development of positive education can be the use of information and communication technologies to improve mental health and psychological well-being. The reported study was funded by the Russian Foundation for Basic Research, project number 18-29-22049.

**Disclosure:**

No significant relationships.

